# Comparação dos Escores HEART, TIMI e GRACE para Predição de Eventos Cardiovasculares Adversos Maiores na Era de Troponina I de Alta Sensibilidade

**DOI:** 10.36660/abc.20200314

**Published:** 2020-05-22

**Authors:** Gabriel Porto Soares

**Affiliations:** 1 Universidade Federal do Rio de Janeiro Rio de JaneiroRJ Brasil Universidade Federal do Rio de Janeiro (UFRJ), Rio de Janeiro, RJ – Brasil; 2 Universidade de Vassouras VassourasRJ Brasil Universidade de Vassouras, Vassouras, RJ – Brasil; 3 Centro Universitário de Valença ValençaRJ Brasil Centro Universitário de Valença (UNIFAA), Valença, RJ – Brasil

**Keywords:** Síndrome Coronariana Aguda, Pontuação de Propensão, Probabilidade, Fatores de Risco, Estudos de Casos e Controle, Troponina/efeitos adverso

As doenças do aparelho circulatório predominam como primeira causa de óbitos no mundo; entre as doenças cardiovasculares, as doenças isquêmicas do coração são o primeiro grupo de causas. A doença cardíaca isquêmica (DIC) é a principal causa global de morte, correspondendo a mais de 9 milhões de mortes em 2016, de acordo com estimativas da Organização Mundial da Saúde (OMS).^1^ A mortalidade por DIC nos países ocidentais diminuiu drasticamente ao longo das últimas décadas, com maior foco na prevenção primária e melhor diagnóstico e tratamento de DIC. No entanto, os países em desenvolvimento apresentam novos desafios para a saúde pública^2^ – tal cenário se reproduz na América Latina. No presente estudo,^3^ realizado na Colômbia, a taxa de mortalidade por DIC era de 150 óbitos por 100 mil habitantes no ano de 2015, representando a principal causa de óbitos naquele país.^4^

Desenvolver escores capazes de prever morte diante das doenças responsáveis pela maior parcela dos óbitos no mundo sempre permeou os objetivos dos cardiologistas. A pergunta “Qual a probabilidade deste paciente com quadro agudo de DIC morrer?” é feita, conscientemente ou não, todas as vezes diante da possibilidade diagnóstica de infarto agudo do miocárdio (IAM) com ou sem elevação do segmento ST ou de um quadro de angina instável.

A busca de variáveis capazes de predizer mortes ou desfechos desfavoráveis – atribuindo ao conjunto destas variáveis modelos matemáticos de probabilidade a curto ou médio prazo – levou ao desenvolvimento de escores, com mais organização e confiabilidade no início dos anos 2000. Iniciou-se com o TIMI (Thrombolysis In Myocardial Infarction Risk Score), para prognóstico e decisão terapêutica nos pacientes com angina instável e IAM sem elevação do segmento ST.^5^ Em seguida, o escore GRACE (Global Registry of Acute Coronary Events), como preditor de mortalidade hospitalar nos pacientes com síndromes coronarianas agudas. O terceiro escore utilizado nessa comparação foi desenvolvido na Holanda em 2007, e é composto por cinco variáveis, formando o mnemônico HEART (**h**istory, **E**CG, **A**GE, ***r****isk factors* e **t**roponina).

A seguir, na Tabela 1, são comparadas as variáveis e as previsões dos três tipos de escores. Chama a atenção o fato de três variáveis serem comuns entre eles: idade, alteração eletrocardiográfica e a presença de positividade em marcadores de necrose miocárdica, especialmente a troponina I. Isso demonstra que essas três variáveis são indicadores independentes de mortalidade e desfechos desfavoráveis em qualquer tipo de síndrome coronariana aguda. O escore GRACE não leva em consideração a presença de fatores de risco ou dados de história clínica, mas, entre os três, é o que contém maior quantidade de variáveis hemodinâmicas: pressão sistólica, frequência cardíaca e classificação de Killip. Uma variável do escore TIMI deve ficar incompleta em grande parte dos casos, pois avalia a presença de uma estenose coronariana prévia; portanto, há necessidade de estudo coronariográfico anterior. O TIMI é o único que também considera qualquer uso de terapêutica antiagregante prévia. No escore GRACE, pode faltar a variável creatinina na avaliação inicial na sala de emergência, pois irá depender do tempo de realização deste exame.

**Tabela 1:** – Comparação das variáveis e previsões de desfechos dos escores TIMI, GRACE e HEART

Escores de Risco		

TIMI	GRACE	HEART
Idade	Idade	Idade
Desvio ST	Desvio ST	ECG: desvio ST – distúrbio inespecífico, repolarização ou BRE – normal
Marcadores +	Marcadores +	Troponina 3×, 1 a 3×, normal
Fatores de risco < 3 ou > 3		FR > 3 ou aterosclerose, 1 ou 2 FR, sem FR
Angina em 24 horas		História clínica
	Frequência cardíaca	
	Pressão arterial sistólica	
	Killip	
Estenose coronária > 50%		
Ácido acetilsalicílico: 7 dias		
	Creatinina	
	Parada cardíaca	

**Previsão de Desfechos**		

**TIMI**	**GRACE**	**HEART**

Previsão para 14 dias: morte, reinfarto, revascularização de urgência	Previsão de mortalidade na internação e para 1 ano	Previsão para 6 semanas de morte, revascularização cirúrgica ou percutânea e IAM

*ECG: eletrocardiograma; BRE: bloqueio de ramo esquerdo; FR: fator de risco; IAM: infarto agudo do miocárdio.*

Os três escores foram construídos para previsão de morte em diferentes intervalos – 14 dias no TIMI; hospitalar e em 1 ano no GRACE; em 6 semanas para o HEART. Vale ressaltar que no estudo comparativo de Torralba et al.,^3^ o intervalo para avaliação dos desfechos foi de 30 dias. Outro ponto a ser criticado é que, no escore GRACE, o desfecho previsto é morte e, em tal estudo, foram analisados os desfechos morte, IAM, revascularização cirúrgica ou percutânea para os três escores, provavelmente reduzindo a sensibilidade do escore GRACE, pois foram analisados desfechos não previstos no modelo matemático preditivo do escore. Vários autores já realizaram comparações entres diferentes escores preditores para doença coronariana aguda, demonstrando desempenho superior do escore HEART^8-10^ em comparação aos outros escores.

O escore HEART é de mais fácil obtenção das variáveis, pois estas são objetivas e presentes no momento do atendimento inicial do paciente; a atribuição de pontuação de 0 a 2 para cada uma das variáveis é mais simples e dispensa uso de calculadoras ou aplicativos para sua obtenção. Tais fatos certamente contribuem para o melhor desempenho na alta sensibilidade de prever eventos cardíacos maiores quando comparados ao TIMI e ao GRACE. Ainda devemos considerar que o desempenho dos três escores foi bem satisfatório para prever eventos, pois até mesmo o GRACE, que demonstrou ser o menos sensível, foi o que apresentou melhor especificidade quando comparado aos outros dois.

Todos os escores apresentam seu papel quando bem realizados, bem aplicados e bem interpretados – lembrando que são valores matemáticos capazes de fazer previsões extrapoladas para grupos populacionais, e não substituem a avaliação individualizada de cada paciente com síndrome coronariana aguda.
