# Supplementary material for: Comparação dos Escores HEART, TIMI e GRACE para Predição de Eventos Cardiovasculares Adversos Maiores na Era de Troponina I de Alta Sensibilidade
Source: Arq Bras Cardiol. 2020 May 22;114(5):803–4. [Article in Portuguese] doi: 10.36660/abc.20200314 (PMC8387002; doi:10.36660/abc.20200314)
Supplement: Translation

# Translation: Comparison of HEART, TIMI and GRACE Scores for Predicting Major Adverse Cardiovascular Events in the Era of High-Sensitivity Assay for Troponin I

**Authors:** Gabriel Porto Soares

**Affiliations:** 1 Brazil Universidade Federal do Rio de Janeiro (UFRJ), Rio de Janeiro, RJ – Brazil; 2 Brazil Universidade de Vassouras, Vassouras, RJ – Brazil; 3 Brazil Centro Universitário de Valença (UNIFAA), Valença, RJ – Brazil

**Keywords:** Acute Coronary Syndrome, Propensity Score, Probability, Risk Factors, Case-Control Studies, Troponin/adverse effects

Diseases of the circulatory system predominate as the leading cause of death in the world; among cardiovascular diseases, ischemic heart diseases are the first group of causes. Ischemic heart disease (IHD) is the leading global cause of death, accounting for more than 9 million deaths in 2016, according to estimates from the World Health Organization (WHO).^1^ Mortality from IHD in Western countries has decreased dramatically over the past few decades, with a greater focus on primary prevention and better diagnosis and treatment of IHD. However, developing countries present new challenges for public health^2^ — this scenario is reproduced in Latin America. In this study,^3^ carried out in Colombia, the mortality rate from IHD was 150 deaths per 100,000 inhabitants in 2015, representing the main cause of deaths in that country.^4^

Developing scores capable of predicting death from the diseases responsible for the largest share of deaths in the world has always been among the objectives of cardiologists. The question “How likely is this patient with acute IHD to die?” is made, whether consciously or not, every time there is a diagnostic possibility of acute myocardial infarction (AMI) with or without ST-segment elevation or unstable angina.

The search for variables capable of predicting deaths or unfavorable outcomes — assigning mathematical models of probability in the short or medium term to these set of variables — has led to the development of scores, with more organization and reliability in the early 2000s. It started with TIMI (Thrombolysis In Myocardial Infarction Risk Score), for prognosis and therapeutic decision in patients with unstable angina and AMI without ST-segment elevation.^5^ Then, the GRACE score (Global Registry of Acute Coronary Events), as a predictor of hospital mortality in patients with acute coronary syndromes. The third score used in this comparison was developed in the Netherlands in 2007 and consists of five variables, forming the HEART mnemonic (**h**istory, **E**CG, **A**GE, **r**isk factors and **t**roponin).

Below, in Table 1, the variables and predictions of the three types of scores are compared. Note that three variables are common among them: age, electrocardiographic abnormality and the presence of positivity in myocardial necrosis markers, especially troponin I. This demonstrates that these three variables are independent indicators of mortality and unfavorable outcomes in any type of acute coronary syndrome. The GRACE score does not take into account the presence of risk factors or clinical history data, but, among the three, it is the one that contains the greatest number of hemodynamic variables: systolic pressure, heart rate and Killip classification. One variable of the TIMI score must be incomplete in most cases, as it assesses the presence of previous coronary stenosis; therefore, previous coronary angiography scan is required. TIMI is the only one that also considers any use of previous antiplatelet therapy. In the GRACE score, the variable “creatinine” may be missing in the initial evaluation in the emergency room, as it will depend on the timing of this scan.

**Table 1:** – Comparison of variables and predictions of outcomes of the TIMI, GRACE and HEART scores

Risk Scores		

TIMI	GRACE	HEART
Age	Age	Age
ST deviation	ST deviation	ECG: ST deviation — nonspecific disorder, repolarization or LBBB — normal
+ markers	+ markers	Troponin 3 ×, 1 to 3 ×, normal
Risk factors < 3 or > 3		RF > 3 or atherosclerosis, 1 or 2 RF, without RF
Chest pain in 24 hours		Clinical history
	Heart rate	
	Systolic blood pressure	
	Killip	
Coronary stenosis >50%		
Acetylsalicylic acid: 7 days		
	Creatinine	
	Cardiac arrest	

**Prediction of Outcomes**		

**TIMI**	**GRACE**	**HEART**

14-day prediction: death, reinfarction, emergency coronary artery bypass grafting	Prediction of mortality at admission and for 1 year	Prediction for 6 weeks of death, surgical or percutaneous coronary artery bypass grafting and AMI

*ECG: electrocardiography; LBBB: left bundle branch block; RF: risk factor; AMI: acute myocardial infarction.*

The three scores were constructed to predict death at different intervals — 14 days at TIMI; hospital death and in 1 year at GRACE; in 6 weeks for HEART. It is worth mentioning that, in the comparative study by Torralba et al.,^3^ the interval of outcome evaluation was 30 days. Another point to be criticized is that, in the GRACE score, the predicted outcome is death and, in such study, the outcomes death, AMI, surgical or percutaneous coronary artery bypass grafting for the three scores were analyzed, probably reducing the sensitivity of the GRACE score, as outcomes not included in the mathematical predictive model of the score were analyzed. Several authors have compared different predictive scores for acute coronary disease, demonstrating superior performance of the HEART^8-10^ score compared to the other scores.

In the HEART score, it is easier to obtain the variables, as these are objective and present at the patient’s first appointment; scoring of 0 to 2 to each of the variables is simpler and does not require any calculators or apps. These facts certainly contribute to the better performance in high-sensitivity prediction of major cardiac events compared to TIMI and GRACE. We must still consider that the performance of the three scores was quite satisfactory for predicting events, since even GRACE, which proved to be the least sensitive one, was the one with the best specificity compared to the other two.

All scores play their role when well performed, well applied and well interpreted — noting that they are mathematical values capable of making extrapolated predictions for population groups and do not substitute the individualized assessment of each patient with acute coronary syndrome.
